# Natural killer cell-based senotherapy: a promising strategy for healthy aging

**DOI:** 10.3389/fimmu.2025.1737572

**Published:** 2026-01-12

**Authors:** Tsutomu Nakazawa, Ryo Yamanishi, Takayuki Morimoto, Ryosuke Matusda

**Affiliations:** 1 Grandsoul Research Institute for Immunology, Inc., Utano, Uda, Nara,, Japan; 2 Department of Neurosurgery, Nara Medical University, Shijocho, Kashihara, Nara, Japan; 3 Social Welfare Organization HIRAKATA RYOIKUEN, Hirakata, Osaka, Japan; 4 Division of Regenerative Medicine, Department of Medicine, University of California, San Diego, La Jolla, CA, United States

**Keywords:** adoptive immunotherapy, antiaging, immunotherapy, NK cell, senescence

## Abstract

One of the most significant risk factors for diseases is aging. Interestingly, some organisms, such as naked mole-rats and most turtles, do not exhibit typical aging-like symptoms or increased mortality as they become older. These aspects indicate that aging is not necessarily an essential event for animal life and are avoidable. Overcoming aging would free humans from age-associated diseases (AADs) and prolong lifespans. Recent studies have demonstrated that one of the causes of age-related organ dysfunction is excessive chronic inflammation caused by the accumulation of senescent cells (SNCs) and their senescence-associated secretory phenotypes (SASPs). Therefore, the development of drugs and medication to remove SNCs is ongoing. Natural killer (NK) cells are integral components of the innate immune system that are critical for clearing SNCs. Beyond this direct function, NK cells also orchestrate innate and adaptive immunity responses to survey and eradicate these compromised cells. Consequently, preserving NK cell function throughout the aging process is paramount for mitigating AADs and promoting robust health in later life. Simultaneously, NK cell-based senotherapy presents compelling avenues for addressing the multifaceted challenges associated with SNC accumulation and aging. Recent investigations into adoptive NK cell-based senotherapy have demonstrated considerable promise in rejuvenating immunosenescence, facilitating SNC elimination. The accumulating evidence provides a promising proof-of-concept for adoptive NK cell-based senotherapy, indicating its potential as a development in longevity therapeutics.

## 1 Introduction

The long-accepted idea of a 120-year maximum human lifespan gained its first statistical validation only in 2016 through innovative artificial data modeling (1). While average human lifespans have increased significantly, the question of maximum lifespan flexibility remains contentious. Research in model organisms has clearly demonstrated that lifespan is responsive to environmental and genetic manipulation (2–4), suggesting that humans might also possess a malleable maximal longevity. Yet, recent findings have controversially indicated that the ascent of the maximum human lifespan may have halted or reversed (1). This re-evaluation of human longevity coincided with a broader scientific challenge to the assumption that aging is a universal, inevitable biological process (5). Critically, species such as turtles (6, 7) and naked mole-rats (8, 9) exhibit “negligible senescence”, defying the typical age-related increase in mortality risk and physical signs of aging. These examples fundamentally bring into question the status of aging as a conserved life characteristic and propose that it could be an intervening, even reversible, pathophysiological state. This review focuses on the burgeoning potential of adoptive natural killer (NK) cell therapy as an innovative strategy to promote healthy aging and longevity.

## 2 Aging and age-associated diseases in humans

The human lifespan has been extended over the past century, but has also created the global challenge of escalating old-age diseases. Aging remains a fundamental, progressive, and irreversible pathophysiological process (10, 11). The idea that aging could be influenced gained early traction with Northrop’s 1925 discovery that light intensity affected *Drosophila* growth and lifespan (2), drawing significant research and public attention. This was reinforced by the fact that caloric restriction delayed age-related conditions and extended longevity in rodent models (3), underscoring the potential for interventions. A pivotal moment occurred in 1983 with the isolation of the first long-lived *Caenorhabditis elegans* strain (4), presenting new frontiers in aging research. Biologists have long contended that aging is critical, yet often overlooked, in the etiology of numerous chronic human disorders (12). Indeed, aging is a recognized risk factor for a wide spectrum of common diseases, including neurodegenerative conditions such as Alzheimer disease (AD) (13–15), Parkinson disease (14, 16), cardiovascular disease (17, 18), chronic obstructive pulmonary disease (19, 20), metabolic disorders such as diabetes (21, 22), and musculoskeletal issues such as osteoporosis (23–25) and osteoarthritis (26, 27). Aging is also a primary contributor to frailty, a geriatric syndrome marked by diminished physiological reserves and increased susceptibility to stressors, stemming from multifaceted biological decline (28, 29). The reality for many aging individuals, especially those aged >60 years, is the management of multiple coexisting health conditions, frequently necessitating complex multi-therapy regimens for effective long-term care (30). Consequently, a profound understanding of the aging process is paramount to uncover novel therapeutic targets and facilitate the future development of clinically applicable pharmacological interventions, addressing the burden of chronic age-related diseases.

## 3 Aging and cellular senescence

Cellular senescence refers to the progressive loss of cellular proliferative and differentiation potential, along with a decline in physiological function over time. Cellular senescence represents a distinct and stable form of cell cycle arrest that is fundamental in tumor suppression in mammals. Beyond its protective effects, senescence exerts major influences on tissue homeostasis and contributes to a range of pathological conditions, rendering it a central driver of organismal aging and aging-associated diseases (11, 31, 32). Although cellular senescence is now recognized as a cornerstone concept in both cancer biology and geroscience, its importance was initially underappreciated. In 1956, cellular senescence was reported as the inability of repeatedly dividing cells to proliferate further when somatic cells derived from normal mammalian tissues were cultured *in vitro* (33). A few years later, in 1961, Hayflick described the finite proliferative capacity of cultured human fibroblasts, an idea that initially faced skepticism from the scientific community (34). However, accumulating evidence over subsequent decades has firmly established senescence as a pivotal physiological and pathological process. Serrano et al. reported on oncogenic-induced senescence (OIS), in which the expression of mutant *Ras* also induces a state of growth arrest similar to replicative senescence (35). In 2005, cellular senescence was reported to function as an important cancer suppression mechanism in precancerous lesions and benign tumors *in vivo* (36–39). Contemporary research continues to contend with challenges, specifically the definition and heterogeneity of senescent cells (SNCs) *in vivo* (40), but the concept is now indispensable, even among its early critics. A growing body of work has elucidated numerous molecular mechanisms underlying senescence regulation, including telomere attrition (41), mitochondrial dysfunction (42), epigenetic alterations (43) impaired proteostasis (44), and stem cell exhaustion (45). Collectively, these features constitute the key hallmarks of aging. Importantly, many of these processes are orchestrated by persistent oxidative stress and elevated reactive oxygen species (ROS) levels, which both trigger and amplify senescence-associated pathways (46).

In normal cells, SNCs are characterized by a stable, stress-induced cessation of the cell cycle in cells that were once proliferative. First documented in cultured human fibroblasts with finite replicative capacity (34, 47), SNCs have since been observed *in vivo*, where their prevalence increases with age across mammalian species, including humans (48–51). These cells exhibit hallmark features such as irreversible growth arrest, upregulation of cell cycle inhibitors such as p16^INK4a^, and activation of stress-responsive signaling pathways including p38 MAPK and NF-κB, that collectively drive the transcriptional reprogramming characteristic of the senescent phenotype (40, 52, 53). This cell cycle arrest is most frequently triggered by a persistent DNA damage response (DDR) or sustained stress signaling, typically mediated through constitutive activation of the p16^INK4a^– retinoblastoma protein (RB) and/or p53 pathways (54). Although SNCs lose their proliferative potential, they remain metabolically active (55) and may preserve certain functional properties of their progenitors. A key driver of the senescence program is activation of the CDKN2A locus, which encodes both p16^INK4a^ and alternate reading frame (ARF). Normally repressed in healthy tissues, CDKN2A is robustly induced by genotoxic or age-associated stress. Notably, p16^INK4a^ expression increases sharply with age and has become one of the most widely used biomarkers for identifying SNCs *in vivo* (49, 56–60).

Another well-recognized hallmark of SNCs is elevated lysosomal β-galactosidase activity, detectable under near-neutral conditions as senescence-associated β-galactosidase (SA-β-gal) staining. This marker was first described in 1995 by Dimri et al., who observed that only senescent, but not proliferating, cells developed a distinctive signal when β-galactosidase activity was assayed at pH 6.0 (48). They introduced a cytochemical assay in which cleavage of the chromogenic substrate X-Gal produces a blue precipitate, enabling the visualization of SNCs. Since then, more refined quantitative methods have been developed to assess SA-β-gal activity at pH 6.0 (61–63). Importantly, this activity reflects the increased expression and accumulation of endogenous lysosomal β-galactosidase in SNCs. However, the enzyme is not required for inducing or maintaining senescence (63). In addition to SA-β-gal activity, SNCs are characterized by the secretion of a diverse array of proinflammatory cytokines, chemokines, growth factors, and proteases, collectively termed senescence-associated secretory phenotypes (SASPs) (64). Although the precise SASP composition varies by cell type and context, its production is largely governed by NF-κB and p38 MAPK signaling and tightly regulated by mammalian target of rapamycin (mTOR)-dependent translational control (65–69). Notably, the SASP has been recognized as a key driver of age-associated tissue dysfunction, highlighting the pathological effects of SNCs on both aging and chronic disease (64, 66). Beyond these features, additional senescence-associated characteristics have been reported, including critically short telomeres, DNA segments with chromatin alterations reinforcing senescence (DNA-SCARS), persistent DDR, NF-κB signaling activation, and the formation of senescence-associated heterochromatin foci (SAHFs).

Cellular senescence is beneficial in tissue remodeling, wound healing, and tumor suppression. However, as individuals age, the accumulation of SNCs coupled with persistent SASP secretion contributes to chronic inflammation, impairs tissue function (31, 70, 71), and fuels development of AADs. The first demonstrations that eliminating SNCs *in vivo* could extend lifespan and improve health were from genetic models (INK-ATTAC transgenic mice) (57). The studies ablated p16^Ink4a^-SNCs using inducible “suicide genes”, and not drugs. The selective clearance of SNCs, known as senolytics, was first successfully tested as pharmacological senotherapy ABT263 (Navitoclax) in a preclinical *in vivo* model, leading to the development of several current senolytic agents (72).

## 4 Immunosenescence

Immunosenescence refers to the gradual deterioration of the immune system that occurs with advancing age, affecting both innate and adaptive immunity. This phenomenon contributes to increased susceptibility to infections, cancer, and reduced vaccine efficacy, while also fostering chronic low-grade inflammation (“inflammaging”), which underlies many AADs (73–75). Yousefzadeh et al. provided a comprehensive and broad overview of the effects of senescent immune cells on cells distributed throughout various organs. They established a conditional knockout mouse by deleting the excision repair cross-complementation group 1 (*Ercc1*) gene, which is needed for DNA repair, only in blood and immune cells. The mice appeared normal when young, but their immune cells aged quickly, exhibiting loss of function and signs of senescence. The aged immune cells then released harmful signals that spread aging to other organs such as the liver, kidney, lung, brain, and muscles, causing tissue damage and decline. Furthermore, transplanting old or *Ercc1*-deficient immune cells into young mice resulted in premature aging in the young mice. Contrastingly, transplanting young immune cells into diseased mice reduced aging signs. Rapamycin treatment improved immune cell function and decreased senescence. The study demonstrated that immune-specific DNA repair defects accelerate immunosenescence, and that senescent immune cells actively drive whole-body aging. Targeting these cells may aid in delaying age-related decline and extend healthy lifespans (76).

Numerous reports have been published on senescence immunity for each cell type. In the adaptive immune system, aging is characterized by thymic involution and a consequent decline in naïve T cell output, leading to restricted T cell receptor (TCR) diversity (77, 78). Additionally, an accumulation of memory and senescent-like T cells, frequently driven by persistent antigenic stimulation such as cytomegalovirus (CMV) infection, further skews T cell repertoires and impairs immune responsiveness (79, 80). Furthermore, B cell compartments undergo profound changes, including decreased generation of naïve B cells, impaired somatic hypermutation, and reduced antibody diversity, which all compromise humoral immunity (81, 82).

In the innate immune system, immunosenescence manifests as reduced function of dendritic cells (DCs), neutrophils, and NK cells, coupled with impaired macrophage phagocytic activity (83–85). NK cells are of particular interest, as both their number and cytotoxic capacity may decline with age, limiting their ability to clear virus-infected cells, tumor cells, or SNCs (86, 87). With aging, macrophages display altered polarization, impaired phagocytosis, and dysregulated cytokine production. Aged macrophages exhibit reduced responsiveness to pattern-recognition receptor (PRR) signaling, including Toll-like receptors (TLRs), leading to impaired pathogen clearance (88, 89). Aged macrophages also exhibit defective efferocytosis (clearance of apoptotic cells), contributing to chronic inflammation and tissue dysfunction (90). Moreover, age-associated skewing toward a proinflammatory M1-like phenotype, along with impaired M2-mediated tissue repair, promotes inflammaging and impaired resolution of inflammation (91, 92). Similarly, DC function is compromised with age. Although total DC numbers may remain relatively stable, their ability to sense danger signals, migrate to lymphoid tissues, and prime naïve T cells declines significantly (93, 94). Aged DCs demonstrate impaired TLR signaling, diminished type I interferon (IFN) responses, and reduced antigen-presenting capacity (95, 96). This contributes to weakened adaptive immune responses, including diminished vaccine efficacy in older individuals (83). Moreover, DCs from elderly individuals tend to produce higher basal levels of proinflammatory cytokines, contributing to inflammaging while simultaneously failing to mount robust protective responses (97, 98). Together, macrophage and DC immunosenescence weaken host defense, impair tissue homeostasis, and exacerbate age-associated inflammation. Collectively, immunosenescence weakens protective immunity and drives a proinflammatory milieu, linking aging with the pathogenesis of cardiovascular disease, neurodegeneration, frailty, and cancer (99, 100).

## 5 NK cells and SNCs

NK cells are bone-marrow-derived innate immune lymphocytes that constitute approximately 10–20% of peripheral blood lymphocytes. NK cells are central to the ability of the immune system to identify and eliminate abnormal cells, including virus-infected cells, tumor cells, and SNCs. NK cells are classified into two subsets: CD56 dim (cytotoxic) and CD56 bright (cytokine-producing). NK cell function is governed by a balance between activating [e.g., NKG2D, DNAX accessory molecule-1 (DNAM-1)] and inhibitory receptors [e.g., killer-cell immunoglobulin-like receptor (KIRs)] (101). SNCs express stress-induced ligands such as major histocompatibility complex (MHC) class I chain-related protein A/B (MICA/B) and CD155, rendering them susceptible to NK cell-mediated cytotoxicity. In addition to direct killing via degranulation (perforin, granzyme B), NK cells orchestrate immune responses through cytokine secretion and crosstalk with T cells and macrophages (13, 102).

**Figure 1:**
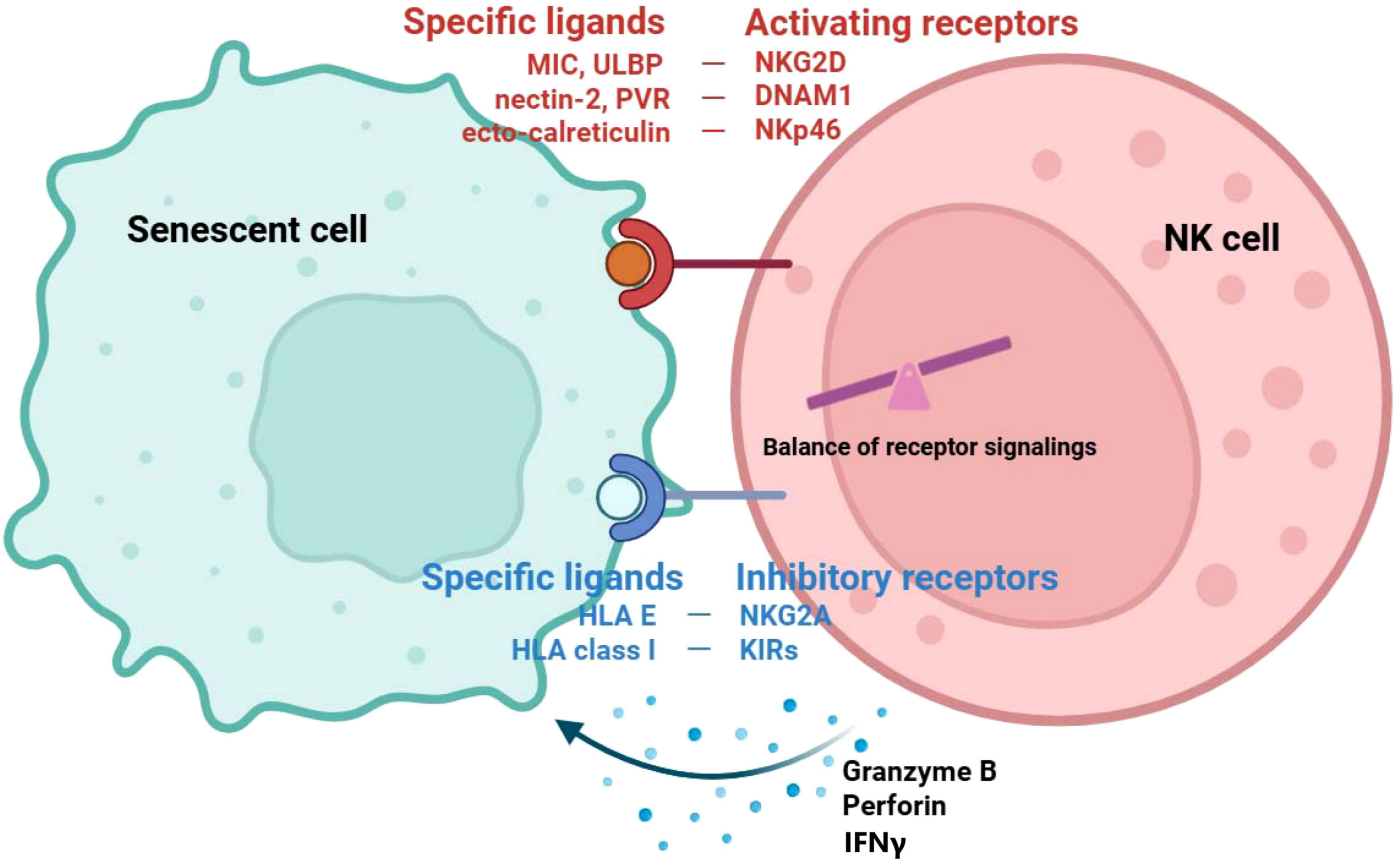
Recognition of NK cell on SNC. NK cells recognize SNCs through a balance of activating and inhibitory receptor signals. Activating receptors such as NKG2D, DNAM-1, and NKp46 engage their respective ligands—MIC/ULBP, nectin-2/PVR, and ecto-calreticulin—on SNCs. Inhibitory receptors, including NKG2A and killer cell immunoglobulin-like receptors (KIRs), interact with HLA-E and classical HLA class I molecules. The balance of these activating and inhibitory inputs determines NK cell responsiveness. Upon recognizing SNCs, NK cells release cytotoxic granules containing granzyme B and perforin, as well as immunostimulatory cytokines such as IFN-γ. Granzyme B and perforin directly induce apoptosis in SNCs, whereas IFN-γ promotes systemic immune activation and could contribute to the clearance of SNCs indirectly. NK, natural killer cell; SNC, Senescent cell.

SNCs display a range of stress-induced surface ligands that render them susceptible to NK cell-mediated clearance. A major pathway involves the NKG2D receptor, which recognizes ligands upregulated during senescence, including MICA, MICB, and UL16-binding proteins (ULBP1–6) in humans, and retinoic acid early inducible 1 (RAE-1), murine UL16-binding protein-like transcript (MULT-1), and H60 in mice (103, 104). These ligands are frequently induced by persistent DDR, oncogenic stress, and ROS (105). SNCs can also be recognized through DNAM-1 and CD226 [T cell immunoreceptor with Ig and ITIM domains (TIGIT)] interactions with its ligands CD112 (Nectin-2) and CD155 [poliovirus receptor (PVR)], which are elevated on senescent fibroblasts and epithelial cells (106, 107). Santara et al. recently reported that NK cells recognize SNCs through the upregulation of stress-induced ligands, including NKp46 ligands. They identified ecto-calreticulin as the long-sought endogenous ligand for NKp46 and demonstrated that NK cells use this pathway to sense endoplasmic reticulum (ER)-stress and SNCs, linking stress responses to immune surveillance (108). NK cells can eliminate SNCs by NK activating receptor recognition via cytotoxic mechanisms, including perforin- and granzyme-mediated killing (103, 109). However, SNCs can develop immune evasion mechanism. Downregulating human leukocyte antigen (HLA) class I molecules in SNCs reduced inhibitory signaling by killer-cell immunoglobulin-like receptors (KIRs) and sensitized them to NK cell cytotoxicity (110). The overexpression of HLA-E, which engages the inhibitory receptor NKG2A on NK cells, has been reported as a strategy for SNCs to escape immune clearance (109). Collectively, these results demonstrate that NK cell surveil SNCs primarily via a balance between activating receptor pathways mediated by NKG2D, DNAM-1, and NKp46 and inhibitory receptor pathways mediated by KIRs and NKG2A (**Figure 1**). But their efficacy declines with age, leading to SNC accumulation and contributing to tissue dysfunction.

## 6 Effects of NK cells on AADs

Historically, immunogerontology research primarily investigated the age-related decline of adaptive immunity (111). An increasing body of evidence now underscores the critical role of innate immunity in the pathogenesis of AADs (70, 102, 106). NK cells have a unique and indispensable position in the innate immune system and are specialized in recognizing and eliminating aberrant cells, such as tumor and virus-infected cells (112–118). Notably, NK cells have also been demonstrated to target SNCs (106, 119, 120) and contribute to immune surveillance by producing cytokines and chemokines, which facilitate the recruitment and activation of other immune cells within the tumor microenvironment (121, 122). Remarkably, research on healthy older adults (those who maintain physical fitness, independence in daily activities, and robust cognitive function) revealed that both the quantity and functional competence of their NK cells were well preserved (123–126). In stark contrast, compromised NK cell function in elderly individuals was associated with a heightened susceptibility to conditions such as atherosclerosis (127) and an elevated risk of all-cause mortality (61, 62). These results underscore that sustaining NK cell functionality is considered essential for promoting healthy aging and contributing to an extended lifespan (106, 128).

NK cells in aging undergo characteristic changes collectively termed immunosenescence. NK cell immunosenescence is manifested by reduced cytotoxicity, impaired cytokine production, and altered receptor expression (84, 87). NK cell subsets are redistributed with age: mature CD56^dim^ cell increase and immunoregulatory CD56^bright^ cells are reduced. Phenotypic alterations include the loss of activating receptors such as NKp30, NKp46, and DNAM-1, alongside an increase in CD57 and NKG2C, frequently influenced by chronic CMV infection (129). Functionally, elderly NK cells demonstrate reduced proliferation and per-cell cytotoxicity, despite preserved antibody-dependent cytotoxicity. Furthermore, cytokine secretion patterns shift, potentially fueling systemic inflammation (84, 130–133). Consequently, SNC clearance declines with age, leading to their accumulation and exacerbation of tissue dysfunction. This impaired NK–SNC axis has been linked to the progression of atherosclerosis, pulmonary fibrosis, sarcopenia, frailty, and cancer (134, 135).

NK cells in AD exhibit complex, and occasionally contradictory, changes. Experimental AD models (triple transgenic mice) exhibited NK alterations before disease onset, suggesting NK dysregulation as an early marker. In humans, the absolute numbers of NK cells in mild cognitive impairment (MCI) or AD are not consistently altered. NK cells also interact with astrocytes and microglia, the main innate immune cells of the brain. Cytokines and complement activation promote NK recruitment across a compromised blood–brain barrier in AD, fueling neuroinflammatory cascades. NK cells may play a dual role in AD: impaired surveillance against pathogens and tumors, but exaggerated inflammatory responses that aggravate neuronal injury. Profiling NK subsets, receptors, and migratory patterns could yield disease progression biomarkers, while therapies targeting NK dysfunction may aid in modulating neuroinflammation in AD (129, 136). These changes contribute to immune evasion by SNCs and subsequent AAD progression, including AD. Lifestyle factors such as exercise and nutrition influence NK cell health, suggesting modifiable pathways to maintain immune surveillance in aging populations (70, 71).

## 7 Evidence for NK cell-based senotherapy

**Figure 2:**
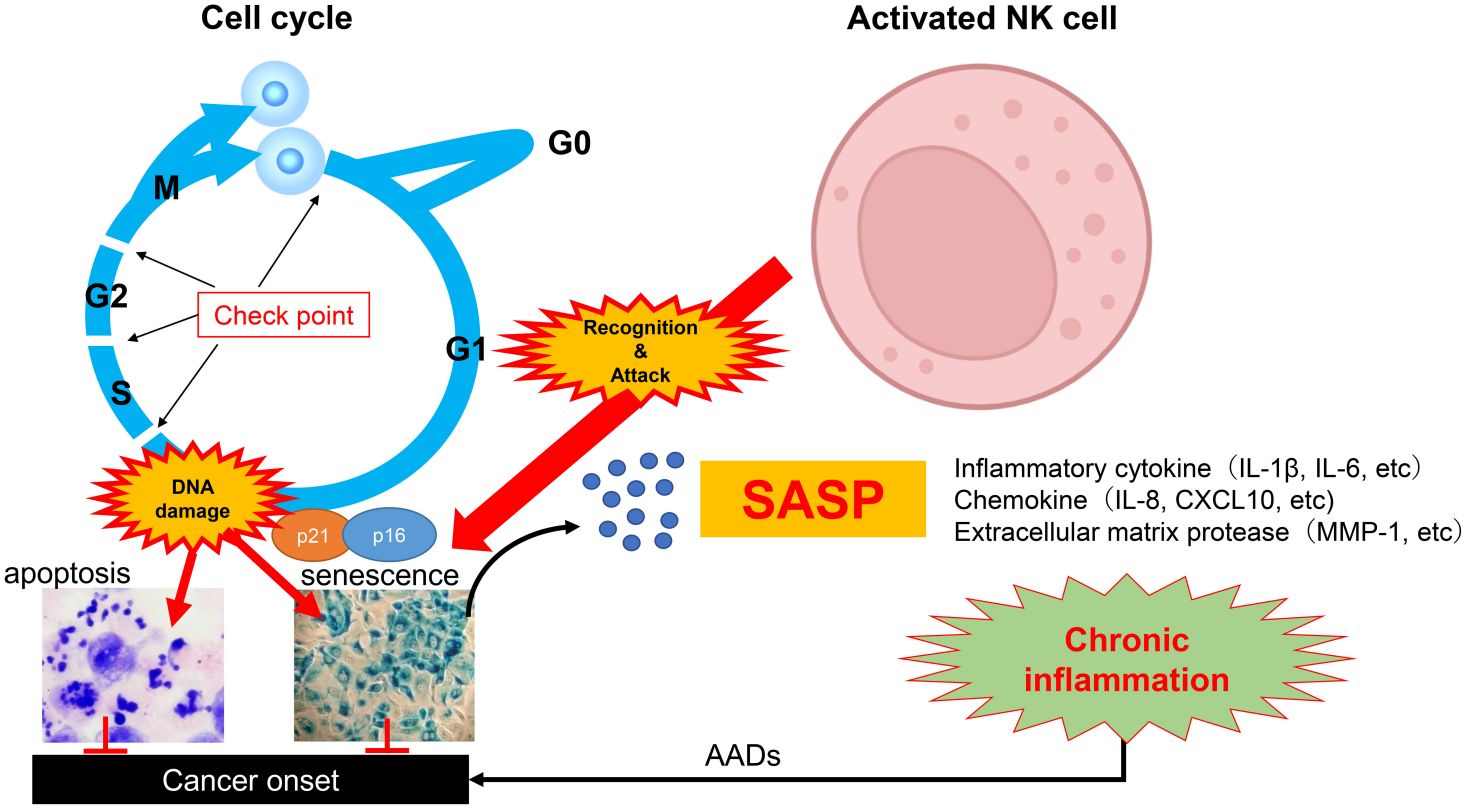
The schematic representation of the potential of adoptive NK cell-based senotherapy for healthy longevity. Cells proliferate through continuous progression of the cell cycle. Upon genomic damage or cellular stress, cell-cycle checkpoint mechanisms are activated, leading to apoptosis or senescence. The induction of senescence is regulated in part by the cyclin-dependent kinase inhibitors p16 and p21. These checkpoint pathways function as critical biological defense systems to suppress malignant transformation. However, with aging, SNCs accumulate in tissues. SNCs secrete SASPs and promote chronic inflammation. Persistent chronic inflammation contributes to the development of multiple disorders including cancers collectively referred to as AAD. Activated and expanded NK cells possess the ability to recognize and directly eliminate SNCs, thereby exerting senolytic activity. NK, natural killer cell; SNC, Senescent cell; SASP, senescence-associated secretory phenotypes; AAD, aging-associated diseases.

In the above context, NK cell-based immunotherapies, especially adoptive NK cell therapy, are drawing considerable attention (**Figure 2**). These therapies have been clinically validated for treating cancers and viral infections (137, 138), and hold promise for reversing immunosenescence, eliminating SNCs, and attenuating the SASP that contributes to AAD pathogenesis (139–141). Among the immune cells, NK cells have garnered significant interest due to their innate capacity to recognize and remove SNCs (70, 71, 102).

The following accumulating results provide a mechanistic basis for NK cell-based senotherapy. Preclinical studies have demonstrated that NK cells can effectively clear senescent fibroblasts, hepatic stellate cells, and pre-malignant cells *in vivo*, limiting fibrosis, tumorigenesis, and chronic inflammation (54, 142). In murine models of liver injury, NK cell depletion led to the accumulation of senescent stellate cells and exacerbated fibrosis, whereas NK cell activity promoted tissue remodeling and recovery (142). Similarly, NK cells delayed tumor initiation by eliminating senescent premalignant cells that otherwise fuel tumorigenesis through SASP-driven inflammation (54, 142). These results highlight NK cell surveillance as an intrinsic senotherapeutic mechanism.

Adoptive NK cell transfer is a therapeutic strategy in which NK cells are isolated, expanded, and occasionally genetically or pharmacologically modified ex vivo, then reinfused into patients to restore or enhance NK cell function. The exploration of various sources for therapeutic NK cells, potentially customizable to target cancer and SNCs, is ongoing (138, 143–145). Recent pilot studies have reported that the adoptive transfer of autologous ex vivo-expanded NK cells reduced SNCs in peripheral blood mononuclear cells (PBMCs) of elderly individuals. In 26 volunteers, autologous NK cell infusion reduced senescence markers (p16, p21) and SA-β-gal in peripheral CD3+ T cells (141). Chelyapov et al. conducted an *in vitro* study using PBMCs from five healthy volunteers, where ex vivo-expanded autologous NK cells were co-cultured with PBMCs. Significant reductions in cells positive for the senescence markers p16 and SA-β-gal were observed post-treatment, with increased expression of activation markers such as CD69 and perforin. The effect lasted several months, but gradually returned to baseline. Repeated infusions prolonged the reduction in senescence markers. Inflammatory proteins such as IL-6, IFN-γ, and MCP-1 decreased post-infusion, while regulated on activation, normal T-cell expressed and secreted (RANTES) increased. No adverse effects or abnormal blood test results were observed (140). A prospective, open-label, randomized controlled trial by Tang et al. enrolled 25 elderly participants who were randomly assigned to receive either autologous NK cell transfusions or no treatment. Over a 90-day follow-up period, the treatment group exhibited a statistically significant decrease in senescent T cell subsets and circulating proinflammatory cytokines, alongside improvements in immune profiling markers. Furthermore, the NK cell transfusion group had decreased exhausted T cells (139). Although these studies are limited in scale and require further validation through larger randomized controlled trials, the results suggest that adoptive NK cell therapy is safe, well-tolerated, and potentially effective in alleviating systemic immunosenescence and reducing the SNC burden in humans.

## 8 Current senotherapy and the advantage of NK cell-based senotherapy

Senotherapeutic strategies are divided into two major categories: senolytics, which selectively induce apoptosis in SNCs, and senomorphics (or SASP modulators), which suppress the harmful proinflammatory phenotype without removing the cells. Numerous preclinical studies have demonstrated that targeting SNCs ameliorates tissue dysfunction, reduces inflammation, and improves health span across various disease models (**Table 1**). Importantly, accumulating evidence now suggests that senolytic and senomorphic strategies are not mutually exclusive, but rather may function in a complementary manner when rationally combined with immune-mediated senescence clearance.

**Table 1:** Current therapeutic strategies targeting cellular senescence.

Category	Agent/Class	Mechanism of action	Main effects	Limitations/Notes
Senolytic	Dasatinib + Quercetin	TK inhibitor + flavonoid	SNCs↓, improved fibrosis & aging dysfunction	Intermittent dosing effective, early senolytic combo
Senolytic	Fisetin	Natural flavonol	p16/SASP↓, renal & muscle function, lifespan extension↑	Orally available, natural compound
Senolytic	Navitoclax	BCL-2/BCL-xL inhibitor	Cleared SNCs in lung, liver, hematopoietic; ↓ fibrosis	Thrombocytopenia (platelets rely on BCL-xL)
Senolytic	Venetoclax	BCL-2 inhibitor	Cleared some senescent lymphoid cells	Limited efficacy since many SNCs rely more on BCL-xL
Senolytic	Digoxin (Cardiac glycosides)	Na^+^/K^+^–ATPase inhibition	SASP↓, fibrosis↓, vascular senescence↓	Preclinical data strong, clinical translation pending
Senomorphic	Rapamycin	mTORC1 inhibition	SASP↓, autophagy, lifespan extension↑	Not directly senolytic, mainly senomorphic
Senomorphic	JAK inhibitors	IL-6/STAT3 blockade	SASP↓, regeneration↑	Approved in myeloproliferative disease
Senomorphic	Metformin	AMPK activation	p16↓, inflammation↓, mitochondrial benefits	Limited direct senolytic potency, more senomorphic
Senomorphic	Statins	Mevalonate pathway inhibition	SNCs↓; slowed arterial stiffness (human data)	Widely prescribed, potential dual cardiovascular-senotherapeutic benefit
Senolytics	SGLT2 inhibitors	Glucose transporter block	senescence markers↓, endothelial/renal function↑	Repurposing from diabetes
Senolytic	Antibody drug conjugates (anti-B2M/uPAR)	Target surface markers by antibody	SNCs↓, dysfunction↓	Preclinical proof-of-concept
Senolytic	CAR-T (anti-uPAR)	T cell-based senolysis	SNCs↓ in liver/lung fibrosis	Early-stage immunotherapy
Senolytic	NKC–based therapy *in vivo* NKC stimulation adoptive NKC transfer	NKC-based senolysis	SNCs↓, SASP↓	*In vivo* stimulation: Safety assessment Adoptive NKC transfer: Manufacturing, donor age, regulatory adaptation

Overview of current senotherapeutic modalities, including small-molecule senolytics, senomorphics, and emerging immune-mediated approaches. Senolytic agents (e.g., Dasatinib + Quercetin, Fisetin, Navitoclax, Venetoclax) eliminate senescent cells (SNCs) by disrupting pro-survival signaling networks such as BCL-2/BCL-xL. Cardiac glycosides and SGLT2 inhibitors have shown context-dependent senolytic or senomorphic properties in experimental models. Targeted biologics and engineered immune cells (uPAR-directed ADCs and CAR-T cells) exemplify next-generation precision senotherapy aimed at selectively clearing SNCs subsets. Senomorphic agents (e.g., Rapamycin, JAK inhibitors, Metformin, Statins) modulate metabolic and inflammatory pathways, attenuating SASP signaling and age-associated tissue dysfunction without directly inducing SNC death. While several agents demonstrate efficacy in preclinical and early-phase clinical studies, challenges remain including tissue-specific SNC heterogeneity, toxicity liabilities, and optimization of treatment regimens to balance efficacy with preservation of beneficial senescence programs.

One of the earliest senolytic combinations combined the tyrosine kinase inhibitor dasatinib with quercetin, a flavonoid with pleiotropic actions. This combination was efficacious in models of idiopathic pulmonary fibrosis, atherosclerosis, and aging-related physical dysfunction (146). Intermittent administration of dasatinib/quercetin reduced SNC burden and ameliorated tissue pathology in multiple organs (146, 147). Fisetin is a natural flavonol with senolytic activity across several SNC types. Fisetin reduced the expression of p16^Ink4a^ and SASP factors in aged mice, improved renal function, and extended lifespan in certain models (148). Furthermore, fisetin benefited muscle strength, mitochondrial function, and reduced tissue fibrosis (149). The BCL-2 and BCL-xL inhibitor Navitoclax induced apoptosis in SNCs by disrupting the SNC anti-apoptotic pathways (SCAPs). Preclinical studies demonstrated that navitoclax effectively cleared SNCs in the lung, liver, and hematopoietic systems, reducing fibrosis and enhancing regeneration (150). However, its clinical development is limited by thrombocytopenia due to BCL-xL inhibition in platelets (151). Venetoclax is a selective, orally bioavailable BCL-2 inhibitor that can clear some SNC subsets (e.g., therapy-induced senescent lymphoid cells), but was less potent than Navitoclax (150, 152, 153). Many SNCs depend on BCL-2 family proteins (BCL-2, BCL-xL, BCL-w) for survival. Many SNC types rely more on BCL-xL than BCL-2. As Venetoclax selectively targets BCL-2, it is less potent than Navitoclax.

Cardiac glycosides such as digoxin have been identified as senolytic agents that target Na^+^/K^+^–ATPase and disrupt ion homeostasis preferentially in human SNCs (154). Digoxin reduced senescence marker expression in murine *in vivo* fibrosis models (e.g. lung fibrosis induced via senescent fibroblast instillation), ameliorated fibrotic histology, and suppressed SASP cytokines (155). Some studies on atherosclerosis models also reported decreases in vascular senescence burden and SASP with digoxin treatment (155), and multiple reviews have cited protective effects in preclinical models of pulmonary fibrosis, atherosclerosis, and type 2 diabetes (156). Originally developed for type 2 diabetes, sodium glucose cotransporter 2 (SGLT2) inhibitors such as dapagliflozin are being repurposed as senomorphic/senolytic agents. Recent studies have demonstrated their ability to reduce senescence markers, improve endothelial and renal function, and suppress SASP components in both diabetic and non-diabetic models (157). Antibody-drug conjugate (ADC) targeting of SNC surface markers such as β-2 microglobulin (B2M) and urokinase plasminogen activator receptor (uPAR), which are upregulated in various SNC types, allows for selective clearance. Preclinical models have demonstrated a reduced SNC burden and alleviated tissue dysfunction without broad cytotoxicity (158).

Chimeric antigen receptor T cells (CAR-T cells) engineered to recognize uPAR or other senescence-specific markers have been efficacious in removing SNCs in models of liver and lung fibrosis. These approaches harness the immune system for targeted senescence clearance (159). As senomorphics and mixed mechanism agents, rapamycin suppresses mechanistic/mTOR complex 1 (mTORC1) signaling, a key regulator of SASP. Rapamycin reduced systemic inflammation in aged mice and progeroid models, preserved organ function, and extended lifespan (160). Furthermore, rapamycin suppressed secondary senescence and promoted autophagy (161). Drugs such as ruxolitinib and momelotinib are used for myeloproliferative disorders, and inhibit SASP by blocking IL-6–STAT3 signaling. In senescent fibroblasts and irradiated tissues, these agents reduced proinflammatory cytokines and improved regeneration (162).

Metformin indirectly reduces SASP through AMPK activation and mitochondrial stabilization. It reduces p16 expression and systemic inflammation in diabetic and aging models, although its senolytic potency is limited (163). Lipophilic statins eliminate senescent endothelial cells by inducing anoikis−related cell death. In human endothelial cell (HUVEC) models of senescence, statins demonstrated senolytic activity (killing senescent endothelial cells while sparing non-SNCs) (164). Fularski et al. reported that statins reduce senescence features in endothelial progenitor cells and other vascular cell types (165). A retrospective human cohort study involved statin users versus non−users in adults with high atherosclerotic risk. Statin use was associated with slower progression of arterial stiffness (measured via brachial−ankle pulse wave velocity), especially in continuous users with high adherence over ~4.8 years (166). Their mechanism may involve inhibition of the mevalonate pathway.

Despite the promise of senotherapeutics, several challenges persist. Cellular heterogeneity among SNCs limits universal targeting. Furthermore, the lack of specific biomarkers complicates *in vivo* detection and treatment monitoring. Potential off-target effects may disrupt beneficial senescence functions (e.g., in wound healing). Translation from mice to humans is hindered by species-specific differences in drug metabolism and senescence phenotypes. Consequently, alternative methodologies capable of safely eliminating a broad spectrum of human SNCs should be investigated. Hence, alternative strategies such as immunological clearance are gaining interest (141). Furthermore, targeting immunosenescence and SNCs has emerged as a pivotal therapeutic strategy for promoting and maintaining healthy aging (111, 167, 168). Compared to senolytics, NK cell-based senotherapy offers greater specificity, reduced systemic toxicity, and the potential for long-lasting effects following a single administration (140). Unlike tumor environments, inflamed tissues with high SNC load support NK cell migration and activation. This localized targeting reduces the risk of off-target effects and enhances therapeutic efficacy.

Importantly, senomorphic agents that suppress SASP and modulate inflammatory tissue microenvironments may further enhance NK cell infiltration, persistence, and cytotoxic activity against residual SNCs. Thus, a combinatorial strategy integrating senomorphic modulation with NK cell-based immune clearance represents a highly rational and potentially synergistic approach for durable senotherapy.

## 9 Biomarkers for NK cell-based senotherapy

The identification of reliable biomarkers is central to the advancement of senotherapies, as they allow for the detection of SNC burden, monitoring of therapeutic response, and prediction of clinical outcomes. Senescence biomarkers are typically divided into molecular, cellular, and functional categories. The following accumulating results support the use of multi-parametric biomarker panels, rather than single readouts, to monitor senotherapy outcomes.

Molecular Biomarkers: The canonical markers include p16^INK4a^ and p21^CIP1^, which are cyclin-dependent kinase inhibitors reflecting stable cell cycle arrest (40, 169). The accumulation of DNA damage foci, such as γH2AX and 53BP1, also indicates a persistent DDR (170). Additionally, the SASP, consisting of cytokines, chemokines, and proteases (e.g., IL-6, IL-8, Matrix Metalloproteinases (MMPs)), is a measurable systemic biomarker linked to age-related inflammation and tissue dysfunction (105). Emerging epigenetic signatures, including senescence-associated DNA methylation profiles, provide complementary information to classical epigenetic aging clocks (171).

Cellular Biomarkers: SNCs are commonly identified by SA-β-gal activity, reflecting enhanced lysosomal content (48). More recently, cell surface molecules have been recognized as actionable biomarkers, including uPAR (159), B2M, and DPP4/CD26, and immune receptor ligands such as MICA/B and ULBPs that engage NK cells (103). These markers allow both therapeutic targeting and immune surveillance. Functional readouts, such as NK cell-mediated clearance capacity against SNCs, are increasingly considered surrogate biomarkers of therapy efficacy (134). Both SNCs and NK cells undergo functional decline with aging or chronic stress. SNCs may downregulate activating ligands or secrete SASP factors that suppress NK cell function (109). In parallel, elderly individuals’ NK cells exhibit impaired cytotoxicity, reduced perforin/granzyme release, and altered receptor expression, diminishing their capacity to clear SNCs (86, 87). This reciprocal dysfunction promotes SNC accumulation and contributes to inflammaging. Functional assays measuring NK cell activity against SNCs are increasingly proposed as surrogate biomarkers of senotherapy efficacy. Measuring NK degranulation (CD107a expression), CD69, perforin/granzyme release, or IFN-γ production provides a readout of functional rejuvenation (141). Ovadya et al. emphasized that NK cell clearance capacity could be a biomarker in senotherapy trials (134). For example, the successful elimination of SNCs by senolytic drugs may be reflected in reduced SASP factors and in restored NK cell effector function. Therefore, NK-mediated clearance assays bridge the gap between molecular markers and systemic outcomes, including improved physical function.

Circulating Biomarkers: Circulating SASP factors, cell-free DNA, and extracellular vesicles reflect the systemic senescence burden (172). Inflammatory mediators such as C-reactive protein (CRP) and TNF-α correlate with age-related SNC accumulation (99).

## 10 Future directions of NK cell-based senotherapy

Adoptive NK cell transfer is an NK cell-based senotherapy. However, further research is needed to optimize NK cell culture conditions, define dosing schedules, and assess the effects of donor age. In particular, donor aging is a critical determinant of NK cell fitness, proliferative capacity, metabolic activity, and cytotoxic function, and may represent an inherent limitation of autologous NK cell-based approaches in elderly individuals. Although ex vivo activation and expansion can partially restore the effector functions of aged NK cells by NK cell stimulation (NK cell activation state), complete functional rejuvenation is not always guaranteed. In this context, the exploration of allogeneic NK cells, umbilical cord-derived NK cells, stem cell-derived NK cells, and CAR-NK cells may broaden clinical applicability. NK cell–based immunotherapies for cancer have been safely evaluated in numerous clinical trials (173). Allogeneic NK cell platforms offer several potential advantages, including superior cytotoxic potency, enhanced proliferative capacity, greater manufacturing consistency, and improved scalability, batch-to-batch manufacturing consistency, and improved scalability, which may be especially advantageous for senotherapy in aged populations where endogenous immune dysfunction is prevalent. From a regulatory perspective, the successful clinical implementation of NK cell-based senotherapy will require strict compliance with Good Manufacturing Practice (GMP) and, in Japan, the Act on the Safety of Regenerative Medicine and PMDA regulatory frameworks. Critical regulatory challenges include the establishment of fully standardized and validated manufacturing processes for NK cell isolation, activation, expansion, and cryopreservation under GMP-compliant conditions, along with rigorous in-process controls and release testing. These quality attributes must encompass cell identity, purity, viability, sterility, endotoxin levels, genomic stability, and functional cytotoxic potency. Moreover, robust and quantitative potency assays predictive of *in vivo* therapeutic efficacy will be mandatory to satisfy regulatory requirements, particularly for allogeneic and gene-modified NK cell products. Process validation, comparability studies following manufacturing changes, and long-term stability testing will also be essential components of regulatory submissions to ensure consistent product quality. With respect to safety evaluation, regulatory authorities will require comprehensive non-clinical and clinical data addressing long-term biodistribution, persistence, off-target cytotoxicity, immunogenicity, and the risk of unintended immune activation. These parameters are of particular importance in the context of repeated dosing regimens and administration to frail elderly individuals with compromised immune homeostasis. In addition, the potential risks associated with tumorigenicity, chromosomal instability, and gene-editing–related off-target effects in CAR-NK or stem cell–derived NK platforms must be rigorously assessed. Finally, large-scale randomized controlled trials will be indispensable to establish the clinical efficacy of NK cell-based senotherapy in extending health span and delaying the onset of AADs. The integration of regulatory science, GMP-compliant manufacturing, and well-designed clinical trials will be decisive for the broad clinical adoption, PMDA approval, and eventual commercialization of NK cell-based senotherapeutic interventions. Addressing these challenges will be crucial to facilitate broad clinical adoption and the development of commercially viable NK cell-based aging interventions.

In addition to adoptive NK cell transfer, therapeutic activation of endogenous NK cells represents an NK cell–based senotherapy. Endogenous NK cells play a central role in the immune surveillance of SNCs through activating receptors such as NKG2D, DNAM-1, and NKp46, which recognize stress-induced ligands upregulated on SNCs. However, aging and chronic inflammation impair NK cytotoxicity and cytokine responsiveness, contributing to immunosenescence. Recent studies have demonstrated that cytokine-mediated stimulation of NK cells, using interleukin (IL)-2, IL-12, IL-15, IL-18, or their combinations, can restore cytotoxicity, enhance IFN-γ production, and generate memory-like NK cell populations with superior effector function (174). This strategy can also be applied for ex vivo activation; however, direct administration of cytokines *in vivo* is likewise conceivable. Nevertheless, systemic cytokine injection carries a potential risk of adverse events, necessitating carefully designed clinical studies to confirm safety. More recently, clinical-grade IL-15 superagonists such as N-803 have been shown to induce robust *in vivo* expansion and activation of endogenous NK cells, illustrating the translational feasibility of this approach (175, 176). Additional strategies to enhance endogenous NK function include checkpoint blockade such as anti-NKG2A antibodies, which release NK cells from inhibitory signaling (177). These findings suggest that pharmacologic enhancement of endogenous NK activity may synergize with senolytic and senomorphic agents to promote more efficient clearance of SNCs. Taken together, these observations indicate that endogenous NK cell activation should be considered a core component of NK cell-based senotherapy, complementing both adoptive NK cell transfer and engineered NK cell approaches. By leveraging the natural distribution and tissue-resident potential of endogenous NK cells, this strategy may facilitate systemic and localized SNC clearance and enhance the durability of senotherapeutic interventions.

Although NK cell-based senotherapy are limited in scale and require further validation through larger randomized controlled trials, the results suggest that NK cell-based senotherapy is safe, well-tolerated, and potentially effective in alleviating systemic immunosenescence and reducing the SNC burden in humans.

## 11 Conclusion

NK cell-based senotherapy represents a promising frontier in preventative medicine targeting the root causes of aging. By leveraging the innate ability of NK cells to eliminate SNCs, this approach has potential to mitigate chronic inflammation, rejuvenate immune function, and improve age-related health outcomes. While clinical translation is in its infancy, the accumulating evidence paves the way for transformative strategies in aging and regenerative medicine.
